# Experimental Research and Multi-Physical Field Coupling Simulation of Electrochemical Machining Based on Gas–Liquid Two-Phase Flow

**DOI:** 10.3390/mi13020246

**Published:** 2022-02-01

**Authors:** Zhaolong Li, Wangwang Li, Ye Dai

**Affiliations:** 1 Key Laboratory of Advanced Manufacturing Intelligent Technology of Ministry of Education, Harbin University of Science and Technology, Harbin 150080, China; lizhaolong@hrbust.edu.cn; 2 School of Mechanical and Power Engineering, Harbin University of Science and Technology, Harbin 150080, China; liwangwang19970214@163.com

**Keywords:** electrochemical machining, gas–liquid two-phase flow, multi-physical field coupling simulation, processing voltage, feed rate

## Abstract

In this paper, the forming mechanism of cooling hole electrolytic machining is studied using multi-physical field coupled simulation and experimental observation. A multi-physical field coupled simulation model was established to obtain the gas–liquid two-phase distribution law inside the machining gap, and a mathematical model of gas–liquid two-phase flow was established to analyze the change law of the size and morphology of cooling hole electrolytic machining under different process parameter conditions. The simulation and experimental results show that the size of the inlet of the cooling hole is larger, the size of the outlet is smaller, and the middle section is more stable; machining voltage and electrode feed speed have a significant influence on the size and shape of heat dissipation holes. Compared with the experimental data, simulation accuracy is good.

## 1. Introduction

GH4169 alloy is widely used in the aerospace industry, for example, in working blades, turbine disks, and the combustion chambers of aerospace engines [1,2,3]. The structure and machining technology of turbine blades directly affect the performance of the engine. In order to improve the power of the turbine engine, and ensure that the turbine works in high-pressure gas above 1 MPa and a high-temperature environment of 1000 °C, it is particularly important to machine the heat dissipation holes of the turbine blades. Because of the small size of the cooling hole structure, traditional machining has higher requirements in terms of bit hardness, and it is easy to break and damage the side wall of the machining hole in the feeding process. However, EDM and laser machining are both hot machining and will inevitably form hot recast layers and microcracks on the metal surface. These issues will affect the machining accuracy, stability and working performance of the heat dissipation holes. Electrochemical machining utilizes the principle of electrochemical dissolution of electrodes in an electrolyte, so that the machined heat dissipation holes have good surface quality, no stress concentration, and no surface hardening layer. Therefore, electrochemical machining technology has a broad application prospect in the field of high-precision manufacturing, such as of thin film heat dissipation holes [4]. Electrolytic machining does not depend on the physical properties of the material, such as hardness, toughness, and mechanical strength, so it is suitable for machining deep holes of small diameter and adding such holes to difficult-to-machine cemented carbides. Electrochemical machining involves many factors, such as electrochemistry, heat transfer, hydrodynamics, etc., and its dissolution and formation state is complex and cannot be directly measured [5,6]. Many scholars have conducted numerous theoretical and experimental studies on the electrochemical dissolution mechanism of materials during electrolytic processing, mainly including simulation analysis of the steady-state process of electrolytic processing of pore structures and experimental and process optimization studies, with good research results being achieved [7,8,9,10,11].

A large number of scholars have studied machining performance and process parameter control and undertaken multi-physical field simulation analysis of electric, flow and temperature fields in the machining gap. They have also undertaken simulation analysis of the combination of convection heat transfer, mathematical modeling and gas–liquid two-phase flow theory, with actual hole structure electrochemical machining, shaping, and machining accuracy [12,13,14,15,16,17]. Through process testing and simulation analysis, the influence of the flow field on the machining gap in machining performance was analyzed, and servo control was optimized, thus improving machining performance [18,19,20].

In this paper, a simulation and an experimental study of the multi-physical field coupling forming process of hole structure electrochemical machining were carried out; a simulation model of cooling hole electrochemical machining was established; and the influence of different process parameters on gas–liquid two-phase flow field and electric field in the machining gap was analyzed. Through comparison of simulation and experiment, the influence of process parameters on the average radius, taper and other response indexes of heat dissipation holes was analyzed, and the accuracy of the multi-physical field coupling simulation model was verified, which is of great significance to multi-physical field coupling research into the process of hole structure electrochemical machining.

## 2. Methods and Experiments

**Figure 1:**
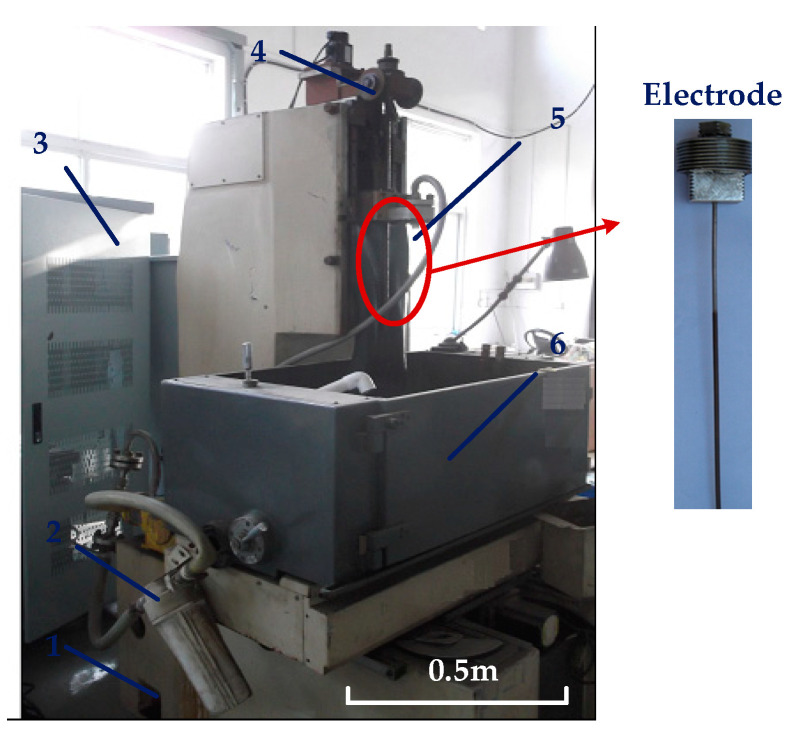
Diagram of ECM equipment processing small hole: 1. filter; 2. pump; 3. control system; 4. motor; 5. electrode; 6. working table.

A schematic view of the machine and processing system for cooling hole electrochemical machining is shown in Figure 1. In the cooling hole electrolytic machining experiment, the tool electrode was connected to the spindle and servo-fed in the Z direction along a motor-driven linear guide. The electrolyte was pumped to the processing area by the pressure in the flow channel, and the tool electrode was clamped by the specially designed fixture in the figure.

The workpiece was GH4169 and the tool electrode was a tubular titanium alloy electrode with an inner diameter of 0.6 mm and an outer diameter of 1.4 mm. Titanium alloy has the advantages of good conductivity, strong corrosion resistance, high strength, good rigidity, etc., and is a good choice for electrochemical machining electrodes (covered with PTFE insulating film). The electrolyte was a sodium nitrate aqueous solution (because the workpiece material was GH4169, a strong acid electrolyte was selected as it has high current density efficiency). Central composite design was adopted and 20 sets of experiments were conducted, with a workpiece thickness of 6 mm, as shown in the process parameters (Table 1). The electrolyte flow rate was 6, 8, 10, and 12 mm/min; the voltage was 12, 16, 24, and 24 V; and the electrolyte concentration was 16. When the machining current was 0, the machining ended. The specific experimental conditions are shown in Table 1.

The main element composition and content of the high-temperature-resistant nickel-based alloy GH4169 are shown in Table 2.

**Figure 2:**
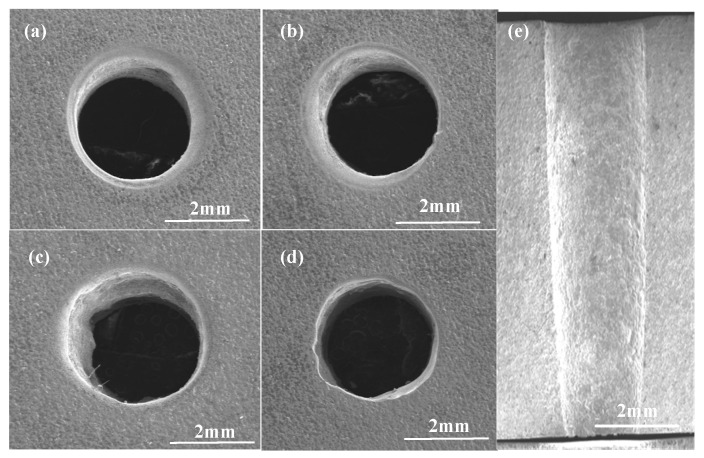
Morphology of cooling hole inlet. The machining speed in (**a**–**d**) is 6–12 mm/min, and (**e**) is the cross-sectional diagram of (**a**).

The entrance morphology of the small hole on the machined GH4169 sample and the sectional morphology obtained by cutting the small hole along the axis by EDM wire cutting were characterized by SEM, as shown in Figure 2. 

**Figure 3:**
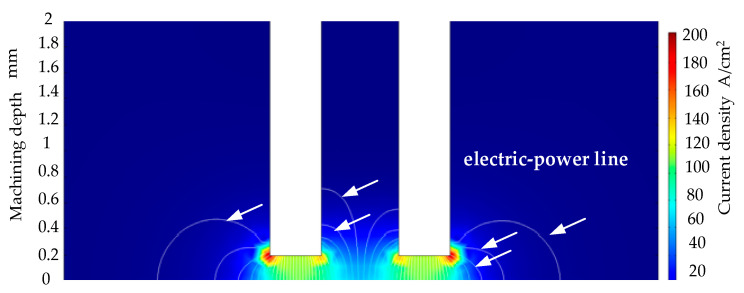
Schematic diagram of electric field distribution in machining gap.

As shown in Figure 2a–e, different machining parameters were used for the electrolytic machining of the heat dissipation holes. The inlet size is slightly different; the inlet shape is better; and the inlet shape is influenced by the electric field in the machining gap. The simulation of the electric field distribution in the electrochemical machining of the tubular electrode is shown in Figure 3. It can be seen that the shape of the air intake is better. Figure 2e shows a sectional view of the cooling hole; it can be seen that the side wall of the cooling hole has a certain taper.

## 3. Results and Discussion

### 3.1. Simulation Analysis of Multiple Physical Fields in ECM Gap of Cooling Hole

**Figure 4:**
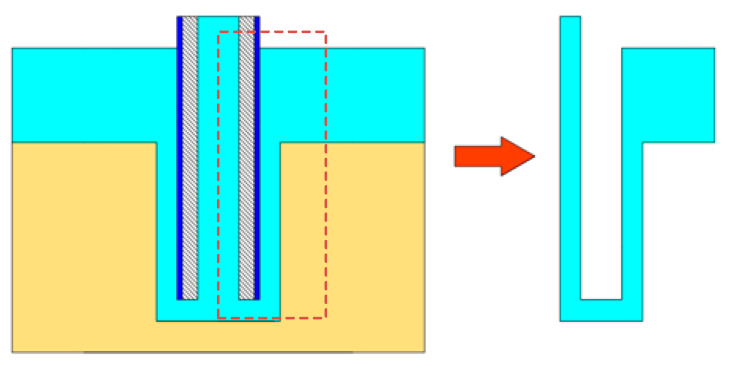
ECM model of cooling hole.

In this paper, the COMSOL built-in drawing tool was used to build a two-dimensional ax symmetric model of the cooling hole. The physical fields analyzed include electric field, flow field and temperature field. The geometric model of the machining gap of the heat dissipation holes is shown in Figure 4.

The specific material simulation parameters are shown in Table 3.

The gap in electrochemical machining contained: (1) hydrogen evolved from the electrochemical reaction; (2) a small number of metal particles, falling off due to the dissolution of the anode; (3) electrolyte. Because the volume of metal particles was very small and had little influence on the conductivity and current density of electrolyte, the gas–liquid–solid three-phase flow in the machining gap was simplified to a gas–liquid two-phase flow.

The electric field model of the machining area was regarded as a constant current electric field and the potential *φ*(*x*, *y*) at any point in the machining gap between the cathode and anode metals satisfied the Laplace equation:

(1) $$\nabla^{2}\varphi =\frac{\partial^{2}\varphi }{\partial x^{2}}+\frac{\partial^{2}\varphi }{\partial y^{2}}+\frac{\partial^{2}\varphi }{\partial z^{2}}=0$$

The electric field intensity *E* is the negative gradient of electric potential *φ*. According to Ohm’s law, the relationship between current density *i*, electric field intensity *E*, and potential *φ* in the machining area is as follows:

E=−∇φ

(2) i=σE=−σ∇φ

*E*—the electric field intensity (V/m); σ—electrolyte conductivity (S/m); φ—potential (V).

See Formula (3) for the normal dissolution rate of the anode workpiece. By solving the displacement of each point on the surface of the workpiece with time, we could obtain the profile of the cooling hole in electrochemical machining at different times.

(3) $$v_{n}=\eta \omega i=-\eta \omega \sigma \nabla \varphi$$

Assuming that bubbles only occupy a small volume fraction and they always move at a free-settling speed, the pressure distribution was calculated according to the mixed average continuity equation. The continuity equation of the gas–liquid two-phase flow model was established (1)–(3):

(4) $$\emptyset_{l}\rho_{l}\frac{\partial u_{l}}{\partial t}+\emptyset_{l}\rho_{l}u_{l}\cdot \nabla u_{l}=-\nabla p+\nabla \cdot \left[\emptyset_{l}\left(\mu_{l}+\mu_{T}\right)\left(\nabla u_{l}+\nabla {u_{l}}^{T}-\frac{2}{3}\left(\nabla \cdot u_{l}\right)I\right)\right]+\emptyset_{l}\rho_{l}g+F$$

(5) $$\frac{\partial }{\partial t}\left(\emptyset_{l}\rho_{l}+\emptyset_{g}\rho_{g}\right)+\nabla \cdot \left(\emptyset_{l}\rho_{l}+\emptyset_{g}\rho_{g}u_{g}\right)=0$$

(6) $$\frac{\partial \emptyset_{g}\rho_{g}}{\partial t}+\nabla \cdot \left(\emptyset_{g}\rho_{g}u_{g}\right)=-m_{gl}$$

where ∅—phase inclusion rate; g—gravity vector; F—volume force (N); $\mu_{l}$—liquid dynamic viscosity (Pa/s); $\mu_{T}$—turbulent viscosity; and $m_{gl}$—gas to liquid mass transfer rate.

Based on the continuity equation of the gas–liquid two-phase model (4)–(6) and actual processing parameters, the distribution law of the gas–liquid two-phase flow field was simulated and analyzed.

**Figure 5:**
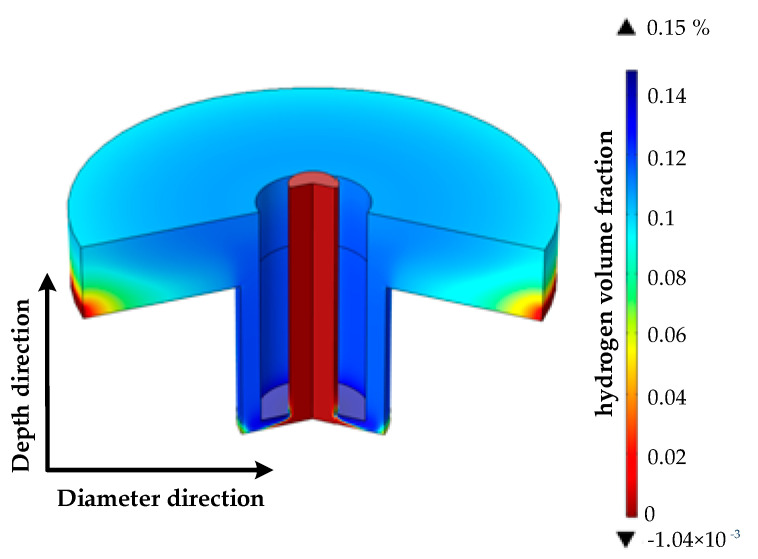
Distribution of hydrogen volume fraction in machining gap.

Based on the above-mentioned modeling analysis, boundary condition setting, process parameter setting and material parameter setting, the simulation analysis was carried out in COMSOL and the conclusion was drawn. The distribution law of the hydrogen volume fraction in the machining gap under the condition of coupling of multiple physical fields is shown in Figure 5.

It can be seen from the figure that the volume fraction of hydrogen gas gradually increased in the direction of electrolyte flow; the fraction of hydrogen gas on the surface of the anode workpiece at the exit point decreased rapidly; and the maximum value was located at the “corner” of the electrode. The maximum hydrogen volume fraction in the machining gap was 0.15. The main cause was that the electrolysis reaction in the machining gap generated hydrogen gas. Due to insufficient electrolyte flow, the volume fraction of hydrogen in the sharp corners and end faces of the tool electrode continued to increase. The volume fraction of hydrogen below the outlet decreased rapidly because of the low density of hydrogen.

This was because the principle of ECM is that under the action of an electric field, an electric current is generated between the electrode and the workpiece, through electrolyte connection; the workpiece is corroded by electrolysis; and hydrogen and oxygen are generated near the workpiece and the electrode. The electrolyte in the machining gap formed between the electrode and the workpiece circulated continuously, in time removing the electrolytic etching products and gas from the machining gap, and thus forming the gas distribution in the electrolyte channel, which conforms to the basic principle of electrolytic machining.

**Figure 6:**
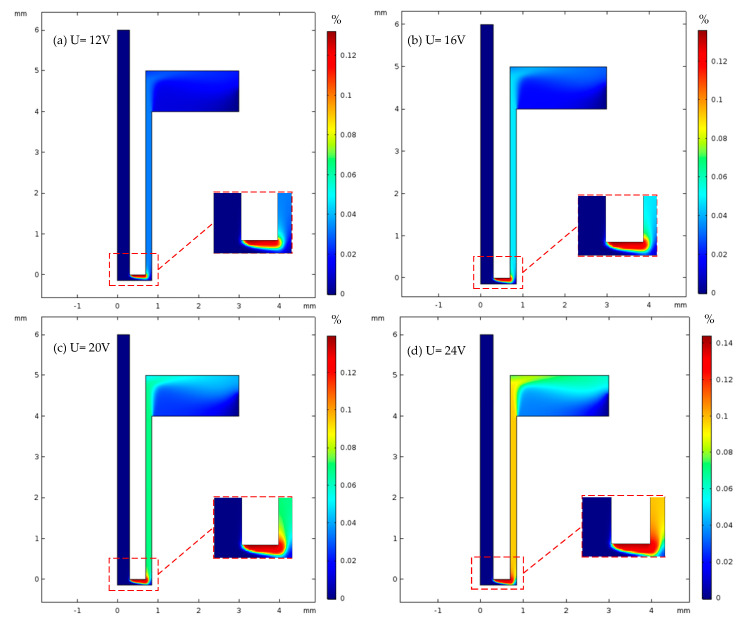
Distribution of gas volume fraction in machining gap of cooling hole with different machining voltages (16% sodium nitrate solution, feed rate 10 mm/min, (**a**) 12 V, (**b**) 16 V, (**c**) 20 V, (**d**) 24 V).

The distribution of the volume fraction of hydrogen in the machining gap of heat dissipation holes under different machining voltages is shown in Figure 6. It can be seen from Figure 6a–d that the hydrogen gas precipitated during the electrochemical reaction accumulated mainly at the end faces and corners of the tool electrode, and the maximum value of the hydrogen volume fraction increased from 0.128 to 0.142 with increasing process voltage. The volume distribution of hydrogen in the end clearance varied greatly, ranging from approximately 0.12 to 0.14.

It can be seen from the figure that when the processing voltage was 12–24 V, the maximum hydrogen volume fractions on the workpiece surface were 0.019 and 0.064, respectively. It can be seen that with the increase in processing voltage, the gas volume fraction increased faster and faster. As the process voltage increased, the volume fraction of hydrogen gradually increased, but the influence range of hydrogen on the surface of the workpiece did not change significantly. When the processing voltage was 12, 16, 20 and 24 V, the maximum values of the hydrogen volume fraction on the workpiece surface were 0.019, 0.027, 0.038 and 0.064, respectively. Under different treatment voltages, the front half of the anodized surface of the workpiece was 0–0.43 mm; it was not affected by the gas and had a zero gas volume fraction. The volume fraction of gas on the surface of the workpiece measuring 0.43–0.7 mm in the middle section gradually increased. The volume fraction of gas decreased in the lower half at 0.7–0.85 mm. The increasing rate of the gas volume fraction increased with the increase in processing voltage. The main reason for this was that the hydrogen gas precipitating from the end face of the tool electrode gradually diffused within the machining gap as the electrolyte flowed under the influence of the tracing force model. Within the range of diffusion, hydrogen bubbles gradually adsorbed on the surface of the workpiece. According to Ohm’s Law and Faraday’s Law, the higher the processing voltage, the higher the current density, and the more hydrogen; therefore, more hydrogen adsorbed on the surface of the workpiece.

**Figure 7:**
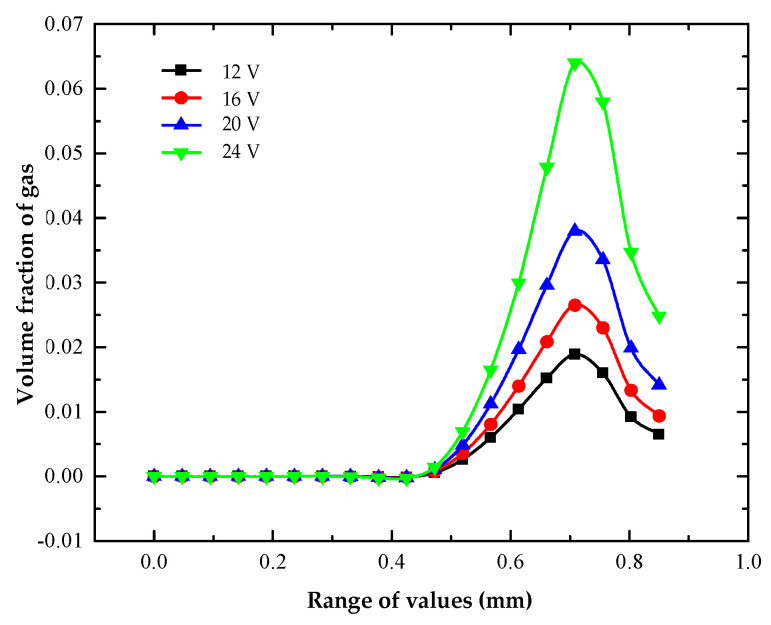
Distribution of gas volume fractions on anodized surface of heat dissipation holes with different processing voltages.

The volume distribution of hydrogen on the machined surface of the heat dissipation holes at different machining voltages is shown in Figure 7.

**Figure 8:**
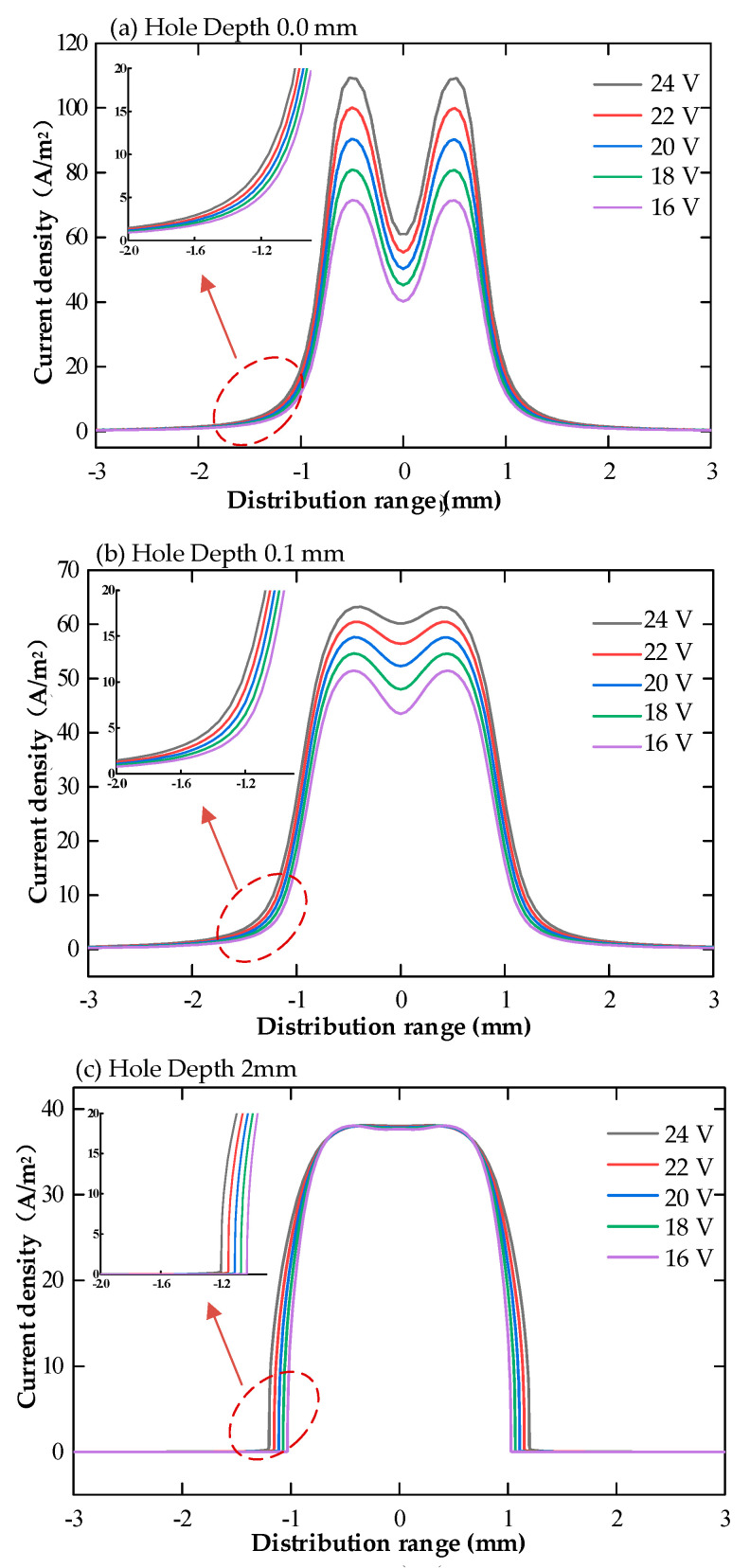
Current density distribution on the workpiece surface at different processing voltages (16% sodium nitrate solution, feed rate 10 mm/min, (**a**) hole depth 0 mm, (**b**) hole depth 0.1 mm, (**c**) hole depth 2 mm).

The magnitude and distribution of current density on the surface of the workpiece for the multi-physics field coupled cooling hole electrolytic machining simulation model without machining voltage conditions was derived, as shown in Figure 8.

**Figure 9:**
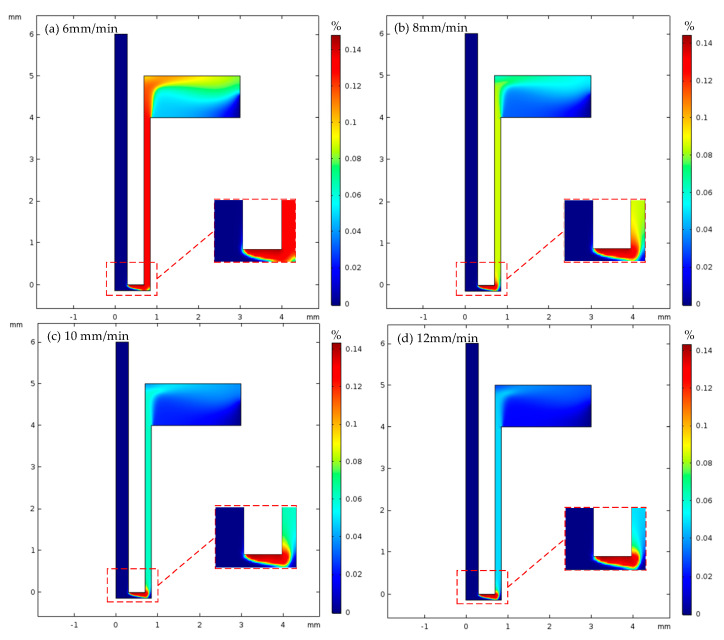
Hydrogen volume fraction in machining gap of cooling hole with different inlet flow rates(16% sodium nitrate solution, U 20V, (**a**) feed rate 6 mm/min, (**b**) feed rate 8 mm/min, (**c**) feed rate 10 mm/min, (**d**) feed rate 12 mm/min).

The distribution of the volume fraction of hydrogen in the machining gap of the heat dissipation holes under different inlet flow rates is shown in Figure 9. It can be seen from the figure that the volume fraction of hydrogen in the machining gap of the cooling hole gradually decreased with the increase in electrolyte inlet velocity. The hydrogen mainly gathered at the end and corner of the tool electrode, and the inlet flow rate had little effect on the maximum hydrogen volume fraction in the machining gap, which was 0.14. The hydrogen distribution in the side gaps was more uniform, while the hydrogen distribution changed more in the end gaps and at the electrolyte outlet. The hydrogen volume fraction in the end gap was also around 0.14.

**Figure 10:**
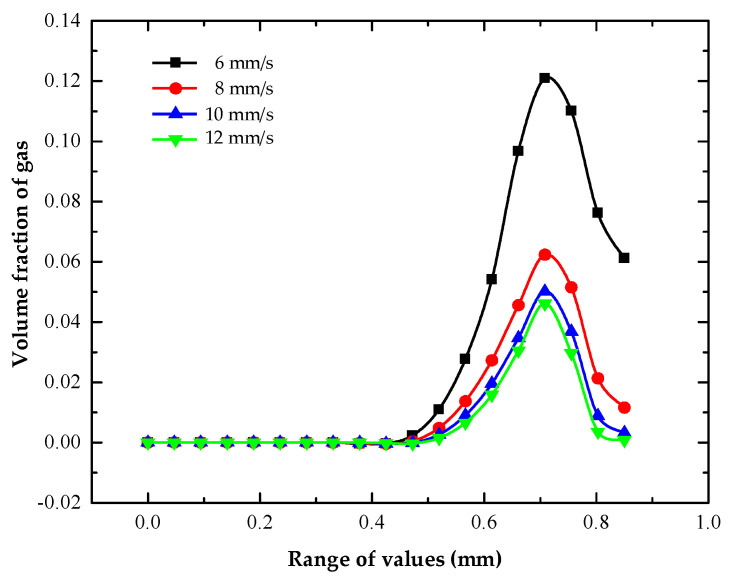
Volume fraction of hydrogen on the machined surface of heat dissipation holes with different inlet flow rates.

The volume fraction distribution of hydrogen on the machined surface of the heat dissipation holes under different inlet flow rates of electrolyte is shown in Figure 10.

When the inlet flow rate was 6 mm/s and 12 mm/s, the maximum volume fractions of hydrogen on the machined surface were 0.121 and 0.046, respectively. At the first half of the workpiece surface (0–0.42 mm), the volume fraction of hydrogen was zero and was less influenced by the inlet flow of electrolyte. In the middle region (0.42–0.7 mm), with the increase in the electrolyte inlet velocity, the growth rate of the hydrogen volume fraction on the machined surface decreased and tended to be stable. In the second half (0.72–0.85 mm), the volume fraction of hydrogen on the machined surface decreased gradually and was less affected by the inlet flow rate. The main reason for this was the increase in the inlet flow rate of the electrolyte and the faster renewal of the electrolyte. This took more hydrogen with it, so the volume fraction of hydrogen on the machined surface was reduced. However, the fast flow rate increased the “vortex” effect and reduced the renewal rate of the hydrogen.

**Figure 11:**
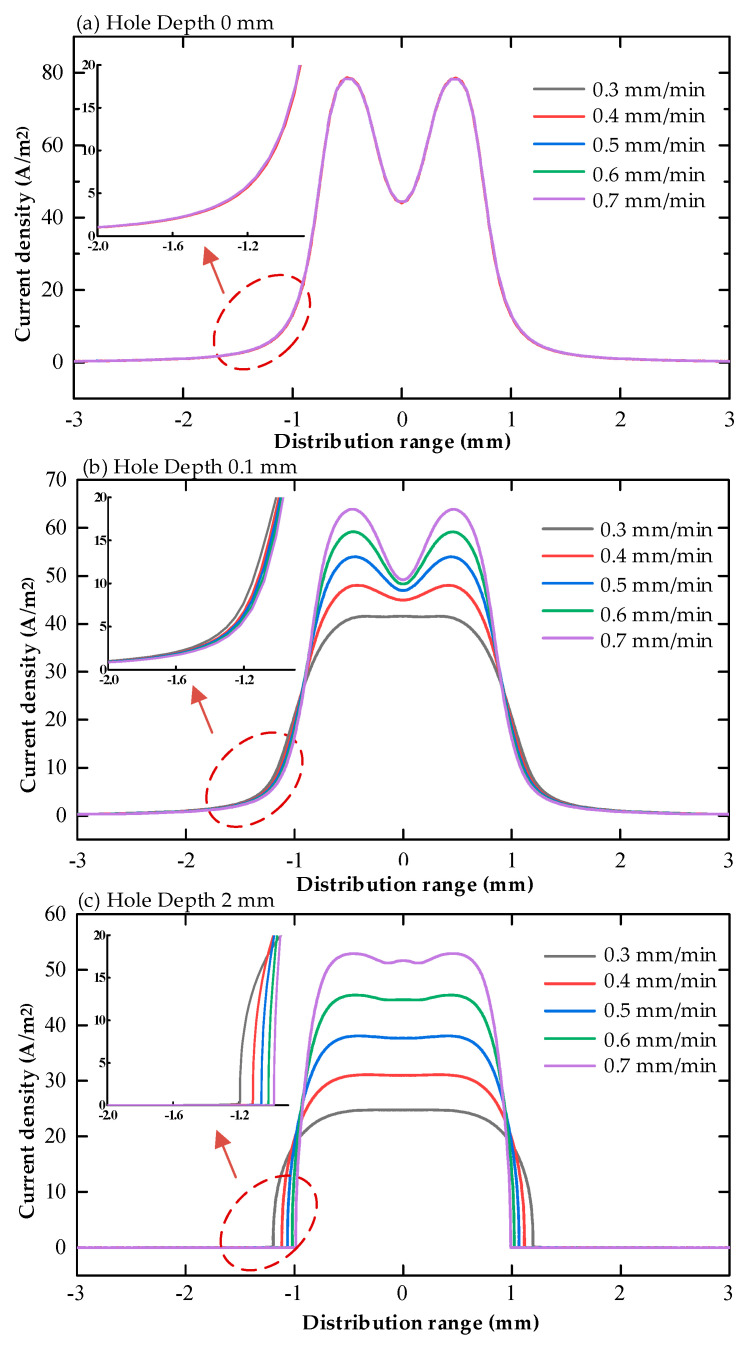
Distribution of current density on the workpiece surface under various electrode feed rates(16% sodium nitrate solution, U 20V, (**a**) hole depth 0mm, (**b**) hole depth 0.1mm, (**c**) hole depth 2mm).

The magnitude and distribution of current density on the surface of the workpiece at different tool electrode feed rates for the multi-physics coupled cooling hole electrolytic machining simulation model was derived, as shown in Figure 11.

**Figure 12:**
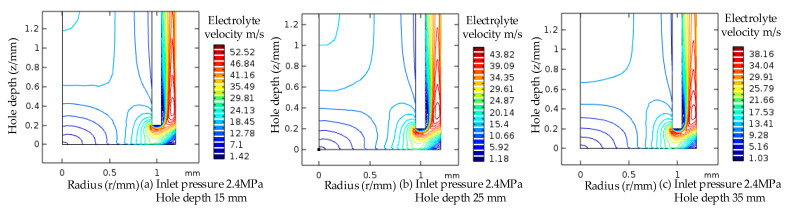
Velocity contour map of electrolyte in the machining gap.

The velocity contour diagram of the electrolyte is shown in Figure 12. The electrolyte inlet pressure was 2.4 MPa. The range of velocity contour values in the same zone was as follows: hole depth 15 mm, contour number 1.18–10.66 m/s; hole depth 35 mm, contour value 1.03–9.28 m/s. The trend of electrolyte flow rate change was basically the same when the processing depth increased; the flow rate difference at the same reference point gradually increased along the radius direction of the low-speed zone.

To sum up, the low-speed zone in the flow field of the processing gap was greatly reduced after increasing the electrolyte pressure, and the flow speed of the electrolyte increased; this removed the electrolytic products and Joule heat in the processing gap in a timely manner and improved the stability and fixed domain of the electrolytic processing.

### 3.2. Analysis of Experiment and Simulation Results

In this part, the simulation model analysis is verified by experiments; the electrochemical machining process parameters and workpieces are the same as the simulation settings.

**Figure 13:**
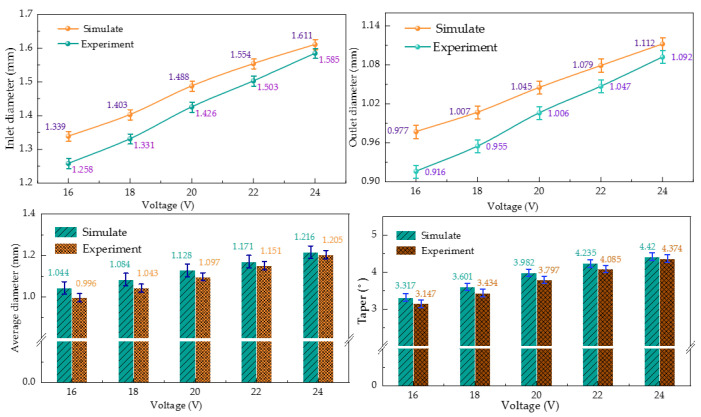
Effect of different machining voltage on ECM size of cooling hole.

The variation trend in the cooling hole size with machining voltage obtained by comparing simulation and experiment is shown in Figure 13. The simulation results show that the inlet radius of the cooling hole was large and the outlet radius of the cooling hole was small. The mean radius and taper of the cooling hole obtained from the simulation were larger than the actual value when the machining voltage was small, and the result obtained from the experiment was larger when the voltage was larger.

Furthermore, the deviation between the experimental and simulated inlet diameters was 4.2%, the deviation between the experimental and simulated outlet diameters was 4.1%, the deviation between the experimental and simulated average diameters was 2.8%, and the deviation between the experimental and simulated tapers was 4%.

The larger entrance radius was due to the large electric field line scattering area and large electrolytic etching range in the initial stage of electrochemical processing, as shown in Figure 3. Therefore, in the incident stage, the range of electrolytic etching was large and the incident radius was relatively large. However, when the electrode continuously entered the workpiece, the machining was at a stable stage, and the electrolytic etching speed and the electrode feeding speed were relatively stable. However, at the exit stage, the distance between the electrode and the workpiece was decreasing, the gap electric field was shrinking, and the electrolytic etching effect became smaller.

**Figure 14:**
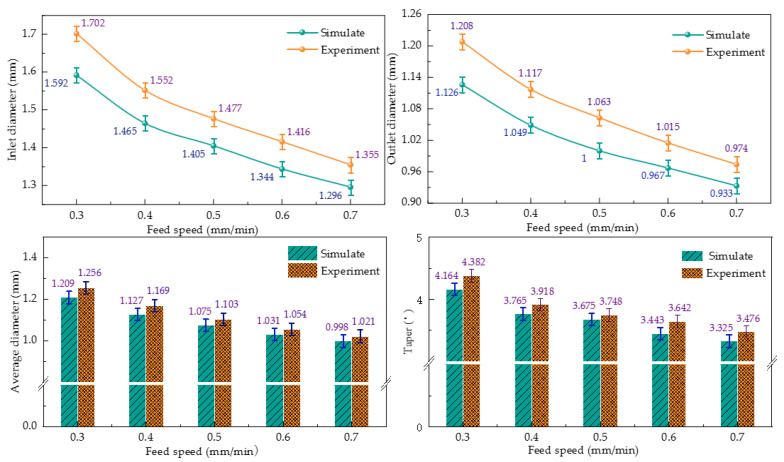
Effect of different electrode feed speed on ECM size of cooling hole.

The simulated and actual values of the electrochemical machining sizes of heat dissipation holes under different electrode feed speeds are shown in Figure 14.

It can be seen from the figure that the size of the inlet radius and outlet radius gradually decreased with the increase in feed speed. The experimental size was slightly larger than the simulation results, and the experimental size increased with the increase in feed speed. The experimental results show that the average radius of the cooling hole was larger and the taper was smaller. The reasons for this phenomenon were the stray corrosion at the inlet and the overfeed at the outlet.

Furthermore, the deviation between the experimental and simulated inlet diameters was 5.6%, the deviation between the experimental and simulated outlet diameters was 5.9%, the deviation between the experimental and simulated average diameters was 2.9%, and the deviation between the experimental and simulated tapers was 4.3%.

## 4. Conclusions

In this paper, the structure of the heat dissipation holes of an air turbine engine was machined by electrochemical machining with tubular electrodes. Compared with other machining methods, electrochemical machining has the advantages of good surface quality of heat dissipation holes, no stress concentration, and no surface hardening layer. In this paper, the theory of gas–liquid two-phase flow in the electrochemical machining of heat dissipation holes was mainly studied, and a simulation model of multi-physical field coupling was established. The dynamic formation of heat dissipation holes in electrochemical machining and the evolution of their size and shape were studied. The variation law of the heat dissipation aperture and taper under different process parameters was verified by experimental simulation. The main research achievements of this paper are summarized as follows.

(1) A simulation model of electrolytic machining of heat dissipation holes was established, and the influence laws of different process parameters on the gas–liquid two-phase flow field and electric field in the machining gap were analyzed. The hydrogen gas precipitated during the electrochemical reaction accumulated mainly at the end faces and corners of the tool electrode, and the maximum value of the hydrogen volume fraction increased from 0.128 to 0.142 with increasing process voltage. The volume distribution of hydrogen in end clearance varied greatly, ranging from approximately 0.12 to 0.14.

(2) The simulation analyzed the influence law of the cooling hole dimensional shape and concluded that the entrance radius and taper of the cooling hole will decrease with the increase in the tool electrode feeding speed and also with the increase in the machining point. When the voltage increased from 16 V to 24 V, the inlet diameter increased from 1.258 mm to 1.585 mm, the sidewall accuracy increased from 3.147 to 4.374, the electrode feeding speed increased from 0.3 mm/min to 0.7 mm/min, the inlet diameter decreased from 1.592 mm to 1.296 mm, and the sidewall taper decreased from 4.382 to 3.476.

(3) Simulation and experimental comparisons were conducted to analyze the influence law of machining voltage and electrode feed rate on the response indexes, such as the mean radius and taper of cooling hole electrolytic machining, and to verify the accuracy of the multi-physics field coupled simulation model. Under different voltages, the deviation between the experimental and simulated inlet diameters was 4.2%, the deviation between the experimental and simulated outlet diameters was 4.1%, the deviation between the experimental and simulated average diameter was 2.8%, and the deviation between experimental and simulated taper was 4%. Under the action of different electrode feeding speeds, the deviation between the experimental and simulated inlet diameters was 5.6%, the deviation between the experimental and simulated outlet diameters was 5.9%, the deviation between the experimental and simulated average diameters was 2.9%, and the deviation between the experimental and simulated tapers was 4.3%.

## Figures and Tables

**Table 1:** Experimental conditions of electrochemical machining of heat dissipation holes.

Experimental Project	Condition
Tool electrode	Titanium alloy tube electrode (covered with insulating film)
Workpiece	GH4169 nickel-based superalloy
Processing voltage	12–24 V DC voltage [12,13]
Electrolyte concentrations	16% sodium nitrate solution
Feed rate	6–12 mm/min [12,13]

**Table 2:** The main elements and content of GH4169.

Element	Ni	Cr	Nb	Mo	Ti	AI	Fe
Atomic mass	59	52	93	96	47	27	56
Percentage	50–55	17–21	4.75–5.5	2.8–3.3	0.65–1.15	0.2–0.8	Margin

**Table 3:** Simulation material parameters.

Simulation Parameters	Values and Units
Liquid specific heat capacity	4200 J/(kg·K)
Hydrogen specific heat capacity	800 J/(kg·K)
Liquid density	1200 kg/m^3^
Hydrogen density	89.9 × 10^−3^ kg/m^3^
Liquid heat transfer coefficient	0.64 [W/(m·K)]
Hydrogen heat transfer coefficient	0.16 [W/(m·K)]
Dynamic viscosity	1.01 × 10^−3^ Pa·s
Bubble diameter	3 × 10^−5^ m
Initial temperature	293.15 K
Temperature correlation coefficient	0.025
Gas correlation coefficient	0.15

## Data Availability

Data are contained within the article.
